# Comparative Genomic Analysis of *Lactococcus garvieae* Strains Isolated from Different Sources Reveals Candidate Virulence Genes

**DOI:** 10.1155/2012/728276

**Published:** 2012-05-09

**Authors:** Eiji Miyauchi, Hidehiro Toh, Akiyo Nakano, Soichi Tanabe, Hidetoshi Morita

**Affiliations:** ^1^Graduate School of Biosphere Science, Hiroshima University, 1-4-4 Kagamiyama, Higashi-Hiroshima, Hiroshima 739-8528, Japan; ^2^Medical Institute of Bioregulation, Kyushu University, 3-1-1 Maidashi, Higashi-ku, Fukuoka 812-8582, Japan; ^3^School of Veterinary Medicine, Azabu University, 1-17-71 Fuchinobe, Chuo-ku, Sagamihara-shi, Kanagawa 252-5201, Japan

## Abstract

*Lactococcus garvieae* is a major pathogen for fish. Two complete (ATCC 49156 and Lg2) and three draft (UNIUD074, 8831, and 21881) genome sequences of *L. garvieae* have recently been released. We here present the results of a comparative genomic analysis of these fish and human isolates of *L. garvieae*. The pangenome comprised 1,542 core and 1,378 dispensable genes. The sequenced *L. garvieae* strains shared most of the possible virulence genes, but the capsule gene cluster was found only in fish-pathogenic strain Lg2. The absence of the capsule gene cluster in other nonpathogenic strains isolated from mastitis and vegetable was also confirmed by PCR. The fish and human isolates of *L. garvieae* contained the specific two and four adhesin genes, respectively, indicating that these adhesion proteins may be involved in the host specificity differences of *L. garvieae*. The discoveries revealed by the pangenomic analysis may provide significant insights into the biology of *L. garvieae*.

## 1. Introduction

The genus *Lactococcus* currently comprises seven species: *L. lactis*, *L. garvieae*, *L. piscium*, *L. plantarum*, *L. raffinolactis*, *L. chungangensis*, and* L. fujiensis* [1]. Among the genus *Lactococcus*, only *L. garvieae* is a major pathogen of fish and causes fatal haemorrhagic septicaemia in fish such as yellowtail and trout [2]. In addition, this bacterium has also been isolated from buffalos with mastitis [3], and even clinical specimens of human blood, urine, and skin [4–6]. Moreover, *L. garvieae* has also been isolated from various kinds of food products including cow's milk [7], cheeses [8–11], meat products [12–14], and sprout [15]. Thus, *L. garvieae* has been considered as an emerging zoonotic pathogen with an increasing clinical significance in both veterinary and human medicine. Although the pathogenic mechanisms of *L. garvieae* are poorly understood, several studies have demonstrated that capsule polysaccharide on the cell surface is one of the virulence factors for fish [12, 16, 17]. Capsulated *L. garvieae* strain is more virulent in fish than noncapsulated one [12, 17, 18]. Nonvirulent *L. garvieae* strains isolated from sprout are also non-capsulated [15].

*L. lactis* is the most studied lactococcal species as a “generally recognized as safe (GRAS)” species, and the genomes of six *L. lactis *strains (IL1403, KF147, MG1363, NZ9000, SK11, and CV56) have been fully sequenced to date. In contrast, the genomic characteristics of *L. garvieae* had not been described, but we recently determined the complete genome sequences of a virulent strain *L. garvieae* Lg2 and a nonvirulent strain *L. garvieae* ATCC 49156 of the fish pathogen [19]. The two strains shared a high degree of sequence identity, but Lg2 had a 16.5-kb capsule gene cluster that is absent in ATCC 49156. The eight genes in the capsule gene cluster were also conserved in several *L. lactis* strains and in the human microbiomes [19]. At approximately the same time, draft genome sequences of other three *L. garvieae* strains (UNIUD074, 8831, and 21881) were also published [20–22]. *L. garvieae* Lg2, UNIUD074, and 8831 were isolated from diseased fish, whereas *L. garvieae* 21881 was from human blood. In the present study, we compared the genomic organization of the sequenced *L. garvieae* strains and also discussed the genes that may be involved in the host specificity of *L. garvieae*.

## 2. Materials and Methods

### 2.1. Informatics

Gene annotation was carried out for each of the draft genome sequences of *L. garvieae *strains UNIUD074, 8831, and 21881 (Table 1). An initial set of predicted protein-coding genes was identified using Glimmer 3.0 [23]. Genes consisting of <120 bp and those containing overlaps were eliminated. All predicted proteins were searched against a nonredundant protein database (nr, NCBI) using BLASTP with a bit-score cutoff of 60. Protein domains were identified by HMMER. Orthology across whole-genomes has been determined using BLASTP reciprocal best hits in all-against-all comparisons of aminoacid sequences.

### 2.2. Bacteria

*L. garvieae* ATCC 43921 (type strain) and ATCC 49156 were obtained from the American Type Culture Collection (ATCC). ATCC 43921 and ATCC 49156 were isolated from mastitis and diseased yellowtail, respectively. *L. garvieae* Lg2 was isolated in 2002 from yellowtail [24]. *L. garvieae *NRIC0607 and NRIC0611 were obtained from radish sprout and broccoli sprout, respectively [15]. These strains were cultured in Todd-Hewitt Broth (Becton, Dickinson and Company) for 20 h at 25°C, suspended in 10% skimmed milk (Becton, Dickinson and Company) solution, and then stored at –80°C until they were used.

### 2.3. Colony Polymerase Chain Reaction (PCR)

To confirm the presence of the capsule gene cluster in *L. garvieae* ATCC 43921, Lg2, NRIC0607, and NRIC0611, a colony PCR was employed. These strains were cultured on Todd-Hewitt agar (Becton, Dickinson and Company) for 20 h at 25°C. The colonies were picked and suspended in the PCR mixture (50 *μ*L) contained 400 *μ*M of each dNTP, 2.5 mM MgCl_2_, 0.05 units of LA-Taq (Takara), 0.5 *μ*M of forward (5′-TGCTGTCATCATATTGTGTCCA-3′) and reverse (5′-GGCTATGGCATTAGTCAGGAAG-3′) primes. The PCR cycling conditions were as follows: 95°C for 3 min, followed by 30 cycles of 95°C for 30 sec, 62°C for 30 sec, 68°C for 5 min, and 72°C for 10 min. After amplification, PCR products and *λ*-EcoT14I digest were electrophoresed on 1.0 gel. The gel was incubated for 15 min in 0.5 *μ*g/mL ethidium bromide for staining.

## 3. Results

Genes were predicted using our own annotation pipeline in this study because the draft genome data of three *L. garvieae* strains (UNIUD074, 8831, and 21881) contain only contig sequences. The general features of genomes of *L. garvieae *strains (ATCC 49156, Lg2, UNIUD074, 8831, and 21881) were summarized in Table 1. The genus *Lactococcus* is included within the family *Streptococcaceae*. We constructed a phylogenetic tree for concatenated sequences of ribosomal proteins from sequenced *Streptococcaceae*, and then *L. garvieae* and *L. lactis* were genealogically distinct (Supplementary Figure 1 available online at doi:10.1155/2012/728276).

**Figure 1:**
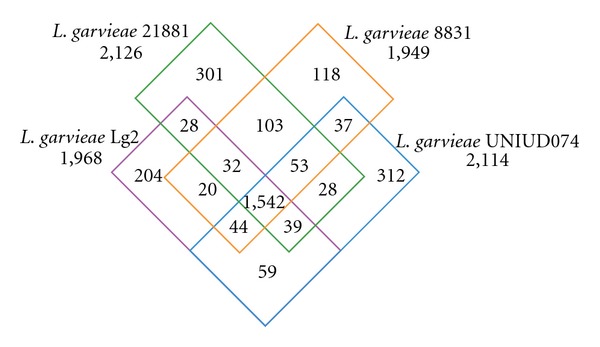
Venn diagram comparing the gene inventories of four strains of *L. garvieae*. The numbers of shared and unique genes are shown.

To identify orthologs shared by *L. garvieae* strains, we produced a four-way Venn diagram (Figure 1). The pangenome consists of 2,920 protein-coding genes with a core of 1,542 genes (53%). A total of 935 genes were found to be specific to the Lg2 (204), UNIUD074 (312), 8831 (118), and 21881 (301) genomes. A majority of the unique genes, in all strains, were annotated as coding for (conserved) hypothetical proteins. The 8831 and 21881 genomes shared most genes (1,730), and this result is in agreement with the phylogenetic tree obtained from concatenated sequences (Supplementary Figure 1). Of the core 1,542 genes, 1,130 (73%) were also conserved among the six sequenced *L. lactis* genomes, suggesting that these genes may constitute the core genome of *lactococci*, likely inherited from their common ancestor.

**Figure 2:**
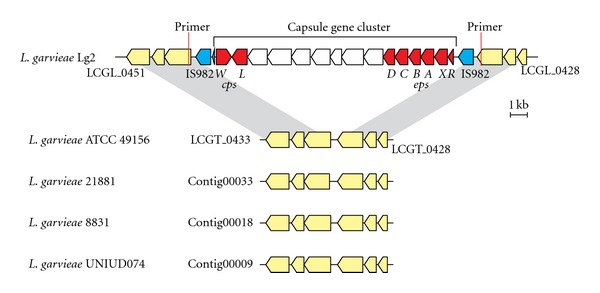
Comparisons of the genomic location of the capsule gene cluster in *L. garvieae* Lg2 and the corresponding location of other *L. garvieae* strains. Genes and their orientations are depicted with arrows using the following colors: red, genes conserved in the capsule gene cluster of various *L. lactis* strains; blue, transposase genes; white, other genes in the capsule gene cluster. Gray bars indicate orthologous regions.

**Figure 3:**
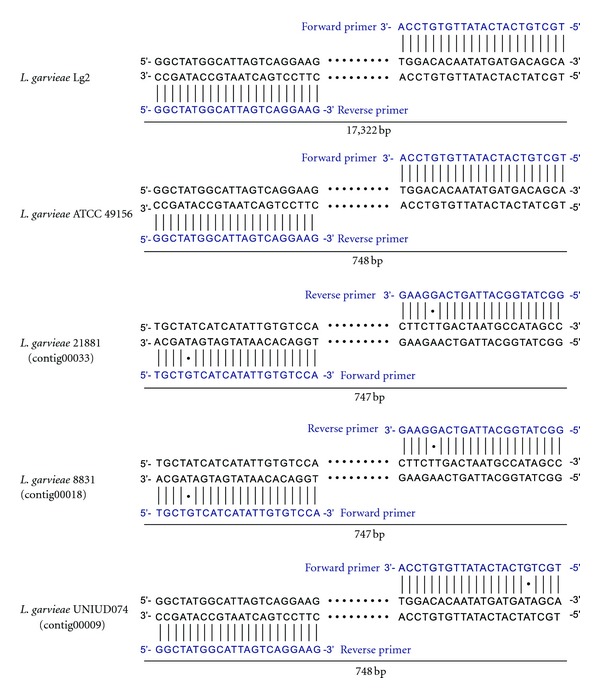
Binding sites of the capsule-specific primers in the *L. garvieae* genomes. The predicted binding sites of the capsule-specific primers in *L. garvieae* strains and the putative PCR products sizes were shown.

**Figure 4:**
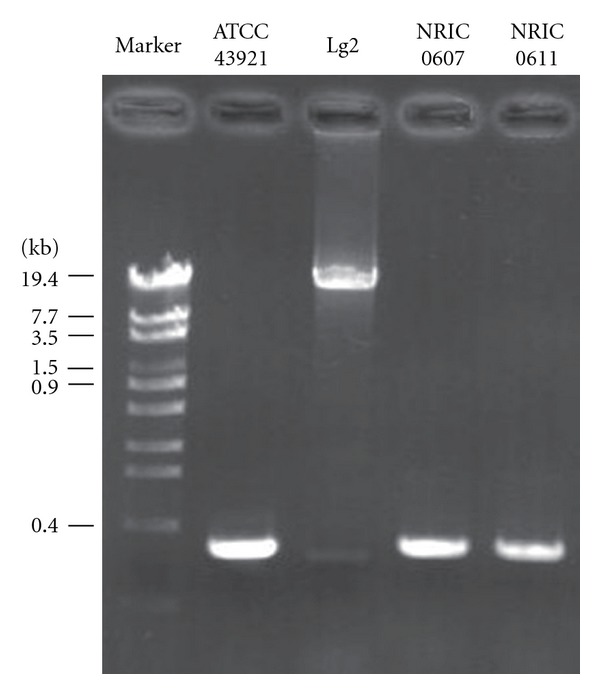
Expression of the capsule gene cluster in *L. garvieae* strains isolated from fish and sprout. The capsule gene clusters in *L. garvieae* strains were amplified by PCR, and the PCR products and marker (*λ*-EcoT14 I digest) were subjected to agarose gel electrophoresis. The gel was stained with ethidium bromide.

The comparative analysis of genomes of *L. garvieae *Lg2 and ATCC 49156 has revealed that Lg2 had a 16.5-kb capsule gene cluster that was absent in ATCC 49156 [19]. The Lg2-specific 204 genes contained all 15 genes encoded in the capsule gene cluster, showing that the UNIUD074, 8831, and 21881 also lack the capsule gene cluster (Table 2). This finding is consistent with the report observed by scanning electron microscopy that *L. garvieae *8831 is a non-capsulated strain (Alicia Gibello, personal communication). Comparison of this genetic locus with the corresponding locus in the sequenced *L. garvieae *genomes revealed that the capsule gene cluster was apparently inserted into the locus syntenic to the sequenced *L. garvieae* (Figure 2). To evaluate the relationship between the virulence and the presence of capsule gene cluster, we next investigated whether nonpathogenic *L. garvieae* strains ATCC 43921, NRIC0607, and NRIC0611 have the capsule gene cluster. The PCR product of ATCC 49156 could be confirmed approximately 750 bp, whose size was as shown in Figure 3, by agarose gel electrophoresis (data not shown). No capsule was observed in *L. garvieae* ATCC 43921, NRIC0607, and NRIC0611, isolated from mastitis, radish, and broccoli sprouts, respectively [15, 24], and they are not pathogenic to fish. ATCC 43921, NRIC 0607, and NRIC 0611, and their PCR products of were approximately less 400 bp, and no capsule gene cluster was also detected in the three strains in the region (Figure 4). The capsule*-*specific primers used in Figure 4 were designed on the basis of the conserved sequences between Lg2 and ATCC 49156, and the binding sites of these primers were found in the UNIUD074, 8831, and 21881 genomes with only one- or two-base mismatches (Figure 3).

Aside from the capsule gene cluster, we have reported other possible virulence genes in the Lg2 genome [19]. Most of the possible virulence genes, such as hemolysin, were also conserved in UNIUD074, 8831, and 21881 (Table 2). However, two adhesin genes (LCGL_1585 and LCGL_1672) are conserved in UNIUD074 and 8831, but absent in 21881. The LCGL_1585 protein contained the collagen-binding domain (Pfam PF05737), and the LCGL_1672 protein contained the mucin-binding domain (PF06458). On the other hand, the 21881-specific 301 genes contained four possible adhesin genes (Supplementary Table 1). The four adhesins contained an LPxTG-type motif, of which two contained the mucin-binding domain (PF06458), and one had a domain (PF05738) conserved in the collagen-binding surface protein of *Staphylococcus aureus*.

No capsule gene cluster was encoded in *L. garvieae* 21881 isolated from human with septicemia (Table 2), and no capsule was detected in *L. garvieae* isolated from human with endocarditis [25]. Other group has reported that *L. garvieae* HF isolated from human contained 23 genes that were absent in *L. garvieae* UNIUD074, using suppressive subtractive hybridization [26]. We evaluated the presence of the 23 genes in the sequenced *L. garvieae* strains (Table 3). Of the 23 genes, 18 were conserved in *L. garvieae* 21881, but only 2–5 were found in the fish isolates.

## 4. Discussion

Little genomic information of *L. garvieae* had been reported, but the complete genome sequences of two *L. garvieae* strains (Lg2 and ATCC 49156) and draft genome sequences of three *L. garvieae* strains (UNIUD074, 8831, and 21881) have recently been released. In this study, the comparative genomic analysis of three fish and one human isolates of *L. garvieae* showed the pangenome structure.

The pathogenic mechanisms of *L. garvieae *are poorly understood. It has only been demonstrated that virulence of *L. garvieae *for fish is, in part, dependent on its ability to form a capsule [9]. Our previous study has revealed that a capsule gene cluster in Lg2 is a genomic island [19]. Supporting these findings, the capsule gene cluster was found only in fish-pathogenic strain Lg2 and was apparently inserted into the locus syntenic to the sequenced *L. garvieae* (Figure 2). ATCC 49156 was originally isolated from diseased yellowtail, has undergone phenotypic changes during its descent from the ancestral strain, and now is nonpathogenic to yellowtails [12, 16, 17]. We were thus tempted to speculate that a common ancestor of ATCC 49156 and Lg2 had acquired the capsule gene cluster before the divergence, and then ATCC 49156 might have been lost the capsule gene cluster during its subculturing.

Several possible virulence genes, such as hemolysin, in Lg2 were also conserved in UNIUD074 and 8831, whereas the capsule gene cluster was not (Table 2). A previous study showed that subculturing of *L. garvieae* in synthetic media resulted in the loss of capsule [15]. Further, we have previously demonstrated that the capsule gene cluster was crucial for virulence of *L. garvieae* for fish [19]. These findings indicate that UNIUD074 and 8831 may be noncapsulated and non-pathogenic for fish.

Although several cases of human infections caused by *L. garvieae* have been reported [1, 24], there is little information about precise mechanisms and factors by which *L. garvieae* causes infection and disease in human. *L. garvieae* 21881 isolated from human with septicemia lacked a capsule gene cluster (Table 2). Other study also showed that no capsule was detected in *L. garvieae* isolated from human with endocarditis [25]. Genes of *L. garvieae* HF isolated from human were also more conserved in 21881 than in the fish isolates. This result suggests that human isolates of *L. garvieae* may share genes that are absent in *L. garvieae* strains isolated from other environments, and that *L. garvieae* strains containing those specific genes, even if non-capsulated, may be able to show pathogenicity in human. Furthermore, the fish isolates of *L. garvieae* shared the two adhesin genes that were absent in the human isolate. Both proteins also contained an LPxTG-type motif for covalent anchoring to the peptidoglycan matrix, and these proteins can be cleaved by sortase. Moreover, Lg2, UNIUD074, and 8831 were isolated from diseased fish, whereas 21881 was obtained from human blood. Thus, the two adhesins may play important roles in the fish isolates because adhesion to host tissues represents a first crucial step in most bacterial infections. Conversely, the human isolate of *L. garvieae* contained the four adhesin genes that were absent in the fish isolates and may be involved in adherence to human cells. These adhesion proteins may be involved in the host specificity differences of *L. garvieae*.

Capsulated strains of* Lactococcus* have been found in fermented milk products. For example, *L. lactis* NIZO B40 isolated from Scandinavian ropy milk has an exopolysaccharide gene cluster, which is similar to that of gram-positive pathogens [27]. As previously reported, capsulated *L. garvieae* strains including Lg2 are avirulent in mice [15], indicating that the capsule would not be a virulence factor for mammalian.

Using the primers specific for the capsule gene cluster, the absence of the capsule gene cluster in ATCC 43921, NRIC0607, and NRIC0611 was confirmed by PCR (Figure 4). These three strains have not shown acute lethality to yellowtail [15, 24], indicating a relationship between the presence of the capsule gene cluster and the virulence of *L. garvieae* to yellowtail. The genomes of UNIUD074, 8831, and 21881 also contained the almost same binding sites of these capsule-specific primers (Figure 3). These results suggest that the PCR analysis using the capsule-specific primers would be a useful method for various *L. garvieae* isolates to determine whether they are capsulated or not.

In conclusion, this study provided insights into understanding of the virulence mechanisms of *L. garvieae* in fish and human. *L. garvieae* has also been isolated from vegetables, cheese, meat, and sausages as nonvirulent strains, and from cow and buffalo as a mastitis agent. The addition of other sequenced strains isolated from various environments will be able to refine the pangenome structure and predict new candidate genes responsible for host specificity of *L. garvieae*, and then the comparative genomic analysis may contribute to the safety of food products containing *L. garvieae*.

## Supplementary Material

Supplementary Figure: Phylogenetic relationships between the genomes of sequenced Streptococcaceae inferred from 27 concatenated ribosomal protein amino acid sequences. The scale bar represents branch length. Reliability of internal branches was assessed using the bootstrap method with 1,000 pseudo-replicates. Bootstrap values greater than 70% are indicated at the nodes. Scale bar represents the number of substitutions per site. An unrooted tree was generated using NJ plot.
Supplementary Table: Sequences of the four possible adhesins specific to *L. garvieae* 21881.

## Figures and Tables

**Table 1:** *L. garvieae* strains used for genome sequence analysis.

Strain	ATCC 49156	Lg2	21881	8831	UNIUD074
Number of contigs	Complete	Complete	91	87	25
Number bases in combined contigs	1,950,135	1,963,964	2,164,301	2,085,932	2,171,472
Number of protein-coding genes	1,947	1,968	2,126	1,949	2,114
Isolation site	Diseased yellowtail^(1)^	Diseased yellowtail	Human blood	Diseased rainbow trout	Diseased rainbow trout
Accession Number	AP009332	AP009333	AFCC00000000	AFCD00000000	AFHF00000000
Reference	[19]	[19]	[22]	[21]	[20]

^(1)^ATCC 49156 was originally isolated in 1974 from diseased yellowtail, and now is non-pathogenic to yellowtails [15].

**Table 2:** Potential virulence factors in *L. garvieae. *

Gene product	Locus	Presence in:^(1)^		
	Lg2	21881	8831	UNIUD074
Capsule gene cluster	LCGL_0431-LCGL_0448	−	−	−
Haemolysin	LCGL_0323	+	+	+
Haemolysin	LCGL_0374	+	+	+
Haemolysin	LCGL_0597	+	+	+
NADH oxidase	LCGL_0664	+	+	+
Superoxide dismutase	LCGL_0285	+	+	+
Phosphoglucomutase	LCGL_1596	+	+	+
Adhesin Pav	LCGL_1330	+	+	+
Adhesin PsaA	LCGL_1533	+	+	+
Enolase	LCGL_1514	+	+	+
LPxTG-containing surface protein	LCGL_1005	+	+	+
LPxTG-containing surface protein	LCGL_1410	+	+	−
LPxTG-containing surface protein	LCGL_1585	−	+	+
LPxTG-containing surface protein	LCGL_1672	−	+	+
Adhesin	LCGL_0196	+	+	+
Adhesin cluster	LCGL_0842-LCGL_0845	+	+	+

^(1)^“+” indicates a locus where a gene is present; “−” indicates a locus where a gene is absent.

**Table 3:** Presence of genes specific to *L. garvieae* HF isolated from human.

Accession Number	Presence in:^(1)^			
	21881	Lg2	8831	UNIUD074
HM852546	−	−	−	−
HM852547	+	−	−	−
HM852548	+	−	−	+
HM852549	+	−	−	−
HM852550	+	−	+	−
HM852551	+	+	+	+
HM852552	+	−	+	−
HM852553	+	−	−	−
HM852554	+	−	−	−
HM852555	+	−	−	−
HM852556	+	−	−	−
HM852557	+	−	+	−
HM852558	−	−	−	−
HM852559	+	−	−	−
HM852560	+	−	−	−
HM852561	−	−	−	−
HM852562	−	−	−	−
HM852563	+	−	−	−
HM852564	+	−	−	−
HM852565	−	−	−	−
HM852566	+	−	−	−
HM852567	+	+	+	+
HM852568	+	−	−	−

^(1)^“+” indicates a locus where a gene is present; “−” indicates a locus where a gene is absent.
