# Supplementary material for: Comparative Genomic Analysis of Lactococcus garvieae Strains Isolated from Different Sources Reveals Candidate Virulence Genes
Source: Int J Microbiol. 2012 May 9;2012:728276. doi: 10.1155/2012/728276 (PMC3357542; doi:10.1155/2012/728276)
Supplement: Supplementary file 1 — Supplementary Figure: Phylogenetic relationships between the genomes of sequenced Streptococcaceae inferred from 27 concatenated ribosomal protein amino acid sequences. The scale bar represents branch length. Reliability of internal branches was assessed using the bootstrap method with 1,000 pseudo-replicates. Bootstrap values greater than 70% are indicated at the nodes. Scale bar represents the number of substitutions per site. An unrooted tree was generated using NJ plot. Supplementary Table: Sequences of the four possible adhesins specific to L. garvieae 21881. [file 728276.f1.pdf]

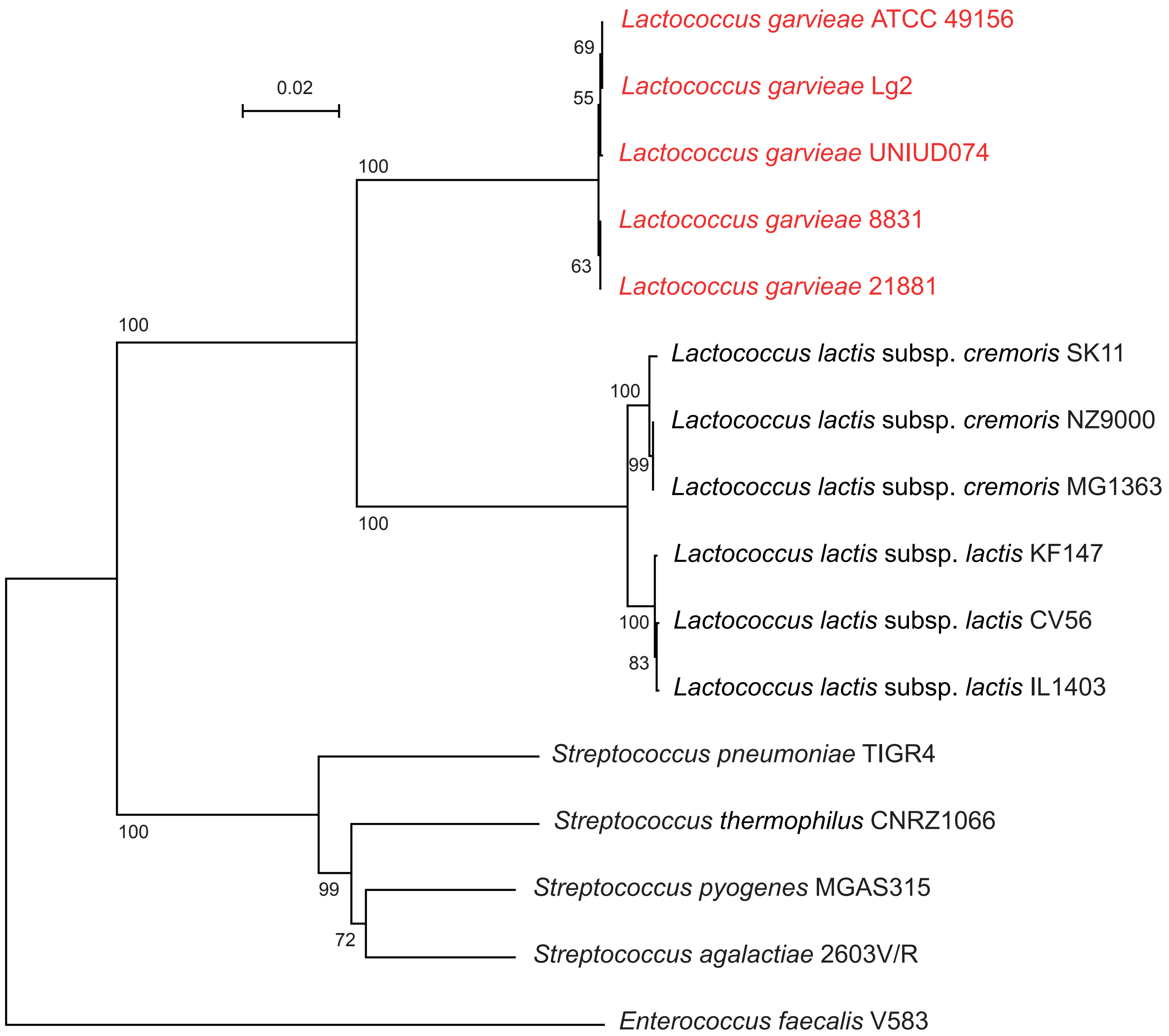

Supplementary Figure 1. Miyauchi et al.

Supplementary Table 1

Sequences of the four possible adhesins specific to *L. garvieae* 21881

>contig00042 complement(5326..8865) 1179aa (PF06458)

MESKNYKKILKSVLVCGAAIMSMTTINALADETEPLQNFTQQMDSMDNTEASNFLRSYGL
RNGTVAGSKVSVSRTSSGFTVQNPKENVESFTANWITDTSKIKLTVSNAFYVNTPIEFSF
KIKQPLAKGEELLIPLNITGTIFNSSSSYTYEGNITQNNGTNYTSQDGSFSYNYDNKTGN
LKVKALDDRGLTDYSGLTLFLNNNWIPVPLRENPMPLKGGVAGNYGAKGSGATVKVKVGD
KETSETLTNTADIDFTTTLQAYNNDIFGKGAEYTTQKTASSSTNFLANFPLINQLKKGKT
KNLLDESMNHYEVKVVSNAKFKNEEDIFKGLSIETYIPIGSSSSGAPITAIAQNATVDKF
PEFKSVIKNASYDKEKQIFSFDLSNKKTDWQALGADLLQNEAYGSSNGKERAAWITDYLK
AMANGNVDQNTTFSSYYPVNSVSFVPVNNNFSLKVDVTVTNSLSKAKTTNHIIVDPVKAG
NGDTTKAAVQLQLMDSEGNPIGELQTPSYYGSGDAYTVTPPVIKGYRLLNVNPQSLIDKL
GITNVVDQQGKFDSSNIGNIYNVVYEYVKEVPLTYSVIDDTTGETLEEHQDLATGALNQE
ANIKENQDKLKALQESYKSKGYLLGNIENGNLPVPTDEKGYNVTLHLSHDAHKAVIDGKS
KTITQTVHYQGAGNETPEDNVQTLTFTSKITQTIDSVTQAVLSEEAPVWSEQQTTAKVTT
PQIKDYEADQAVVEGSTFTHDNSDKKVTVTYKSTLAPLTYTIVDDTTDKTLEKEVDFAMG
TVGEEVNTSDNQEKLKQLIKSYTDKGYLLGNSENADLPTPKDNTGYTITLHFTHATHEQV
VDGETKKVTQTIHYQGAGNETPEDDVQVLTFTSKITQTIDNVTHRVLAEAKPVWPEQQST
RAVDSPTLKDYAADKESVPSMNYNYDSEDVVLEVNYSSTIAPITYSVIDDTAGKTLEDKK
DFVMSHVLEPINTSENQDKLKSIAKSYTDKGYVLGNIENEELPVPGDTKGYDVVFHLTHG
TSEKVIDDESKTVTQKIQYKGAGAKTPEEDIQKFVFTSTLTQTIDDVTGDVVSTSGPTWS
DNQHSKAVKTPEIDGYSSDIDTVKSYDYDHEAKDKTITVSYKEVPPVVKEVKKVVKNILP
QTGTEAEIGLAFTGAALVLIAGALYVKSKLRKESIKNDD

>contig00042 complement(39668..43489) 1273aa (PF05738)

MKKQHKIRQLTKALTEKKLSHFSMRKSGKRWITRSAIVLALAGGTGLIAVINPSLISTVA
YALTLPPGNTQIGSIGNAPVIQTSNNNFSTMQPLFDSLTGTIQPEYIVGDTQDGTGGKPV
AWNSSTTYAGTAISNANSLVNGANGLSYDTIHYGMGISDGFQDNDKFNYVSKGQEINIKN
IGTAYNVKTGKNVPIGMAVKINDATYYDSTTSSSPRNVFGDGFRLLVGARNNGGTITLGY
MVSMDGIPNSGSGQGNEGGGSGGSGGDAIGAASGIPESVRGTISLYDMDTKQNLPASTIM
AMKVSDIDAGQSAQLSNGALGYIVSKPTNLELRDNILTAKGNDTVVGDSANLNANSYIAL
YNGSSTALNFVDTNGNQGQGSIIQAVFGNLGSTSPHQSLGYIEIDKTTIQYGKDLPNNLY

DFQDISFDVLDSTGKKVDTLKLDSSGKSVKSKGLPPGKYTLHETSGKWSSTGQTVLPDFT
VEVKAGDTTTAKPKNTAVTGEITIKKSGVESGDAMWNENYTLEGTTFKLTSKTDGKTYTA
VIGKESNGEWKTTVKDLPLGTYTIEETKASPGFTNTFEKKEVTLSYKDQHTEIVFGETKG
TNQEVKGENTLKKSDNETGTDQNGKGVLKTAKYAYFHDDDSTGSSPHKKGDPVKWSEKPA
PKLLAGEVVHSAIIGGNPVSYGDNVVIEIDDESLSIALGNLPAGKYKVREVDAGEGYVTD
PTEHKFEIQKQDDKTQNIVTPESSSKEQLIKAKITLDKMVTLPDSQGGSGYNGIEFTAEP
MEGTVADPVKFVTGVNPNTGDDGYASQELAYGDWKVSETKGVEGYEDVKPVYIHMETDTE
KDILTISASYYEDFSKPFSKRTFSLKDSSTESNPNGEGTVGEVTPEVPTISLSTIHFNDN
PTPEKVPSIDIEKANDKIPEPGEGNHKDKDNNGGENDRDTPDTALEVESGKSTAIKFRFT
NNGTEALTHLKVSDKTIEGGISIKDPAWFFNGEKLSTNKDGELVTKDGKLLVLQPDEFVT
GEGTLPELSDGKLHGDEVSVSGVGVESGKEVGDEDKWYGKPKEEVPSIDIEKADSKAPEA
GEGNHKDKPNNAGENDHDTEDTAKELEENKATEIFFKTTNNGTEPLTHLKIVDKTIAGKA
NVKNIKLTYKEKPLKVNKDGELTTEDGKLLVLNPGETIEGKGTLEGLAAGDIHGDNVTVD
GVGVHSGKKVGDDDNWYGKVTPKTPEQPKNNIFNISLPKTGMKAQTILTAIAAVIVVIVA
GLGTFFYRKNKMK

>contig00026 complement(51658..52068) 136aa (PF06458)
VTFTRQVSTDTVTGEKTYGSWSADQSFAAVTSPVIKGYTPDQAEIGAQTVSGDASDLDFT
VIYTKDAPTKPVNPSQPTTPAKPVQAGQAAATNFVDQRLPQTGETDQQHMTLSGLLLLAM
SSVLGLFGMTKRQRKE

>contig00049 complement(2350..3180) 276aa
MKKTLIAGLILVTVSIGVQASADSINGYQVTLTDSNTAIGKNGLIWHNDGGTWYADDGSE
NGFIAPDKPEIPAIKTPKIPESTAKPIVPHKVLPEQKIPTKLLTVDTTDNIPGEVVNVPQ
HIEQLTPPNPNVGAHLDAPQHIEKAPKPIKQNIPEPIAVKNLTEKPKINKNSSSTVRIIE
PKKLDNSVTHYSIPSDTEQEIKKAKNDTHVATLTPKTQAVLTTLGTGMKSSPTRTYQAKG
TPLPATGEDDTMTYVVLGMSSIVGAAVLALYLQRNS

* The LPxTG-type motifs are highlighted in blue.
